# ‘Elective caesarean section at 38–39 weeks gestation compared to > 39 weeks on neonatal outcomes: a prospective cohort study

**DOI:** 10.1186/s12884-018-1785-2

**Published:** 2018-05-08

**Authors:** Reihaneh Pirjani, Motahareh Afrakhteh, Mahdi Sepidarkish, Shahin Nariman, Mahboobeh Shirazi, Ashraf Moini, Ladan Hosseini

**Affiliations:** 1 Obstetrics and Gynecology Department, Arash Women’s Hospital, Tehran University of Medical Sciences, Tehran, Iran; 2 Research development center, Arash Women’s Hospital, Tehran University of Medical Sciences, Tehran, Iran; 3 School of medicine, Tehran University of Medical Sciences, Tehran, Iran; 4 Department of Epidemiology and Reproductive Health, Reproductive Epidemiology Research Center, Royan Institute for Reproductive Biomedicine, ACECR, Tehran, Iran; 5 Department of Pediatrics, Arash women’s Hospital, Tehran University of Medical Sciences, Tehran, Iran; 6 Maternal, Fetal and Neonatal Research Center, Tehran University of Medical Sciences, Tehran, Iran; 7 Obstetrics and Gynecology Department, Arash Women’s Hospital, Tehran University of Medical Sciences, Tehran, Iran; 8 Department of Endocrinology and Female Infertility, Reproductive Biomedicine Research Center, Royan Institute for Reproductive Biomedicine, ACECR., Tehran, Iran; 9 Research development center, Arash Women’s Hospital, Tehran University of Medical Sciences, Tehran, Iran

**Keywords:** Caesarean section, Intensive care units, Neonate, Transient tachypnea of the newborn, Respiratory distress syndrome

## Abstract

**Background:**

This study was conducted to compare neonatal complications in scheduled cesarean sections (CS) between 38 and 39 gestational weeks with CS performed after 39 gestational weeks in Iranian low -risk pregnant women.

**Methods:**

In this cohort study, 2086 patients were enrolled based on the inclusion and exclusion criteria. The neonates were evaluated in terms of the following items: transient tachypnea of the newborn (TTN), respiratory distress syndrome (RDS), sepsis, need for NICU hospitalization, birth weight, birth height, head circumference, and the first minute and fifth minute Apgar score. Several multiple logistic regression models were performed for each response variable (adverse outcome) separately.

**Results:**

The incidence of NICU admission was significantly higher in neonates born at 38–39 gestational weeks than those who were born after 39 gestational weeks. No significant differences were found in the incidence of neonatal sepsis, TTN, and RDS between the two groups.

**Conclusion:**

According to our study results, elective CS at 38–9 weeks’ gestation is associated with a higher rate of TTN and NICU admission in comparison with elective CS performed after 39 completed gestational weeks.

## Background

The appropriate gestational age for scheduled cesarean section (CS) has become a topic of interest in prenatal care [1]. For the past four decades, obstetricians and pediatricians, assuming that fetal maturity could be completed at the end of 37 gestational weeks, have defined “the term pregnancy” from that time onwards [2]. However, it has now become clear that even in “the term pregnancy” (37 gestational weeks), neonatal respiratory complications will decrease with an increase in the gestational age to 39 weeks. These findings have challenged the old definition of “theterm pregnancy” [3]. Recently, the definition of “the term pregnancy” has changed to cover three categories of premature term (37–38 weeks + 6 days), full term (39–40 weeks and 6 days), and late term (41-41 weeks + 6 days) [4].

Experts recommend that scheduled cesarean delivery be conducted from 39 weeks onwards [5], so that fetal maturity is complete. Some studies have shown discrepancies in respiratory complications according to the gestational age pattern between Asian and Caucasian ethnicities. The lowest complication rate was observed in 39–40 gestational weeks in the Caucasians and in 38 gestational weeks in Asians [1]. Accordingly, this study aimed to compare neonatal complications in scheduled CS between 38 and 39 gestational weeks with scheduled CS performed after 39 gestational weeks in Iranian pregnant women.

## Method and materials

This prospective cohort study was conducted at a university hospital (Arash Hospital, Tehran, Iran) from April 2013 to April 2015. All term singleton pregnancies (≥38 gestational weeks) scheduled for elective CS were enrolled in this study. The research protocol of the study was approved by the Ethical Research Committee of Tehran University of Medical Sciences. The informed consent was obtained from all pregnant women. The inclusion criteria were singleton pregnancy and elective CS scheduled for 38 complete gestational weeks or later. The exclusion criteria were gestational age below 38 completed gestational weeks, multiple pregnancy, maternal chronic diseases, gestational diabetes, preeclampsia, CS due to fetal distress, intra uterine growth restriction, meconium defecation, placenta previa or accreta, fetal abnormalities, CS during the active phase of labor, repeated CS after beginning of uterine contracture, and any other cause of emergency CS.

All patients had two ultrasonography reports in the first trimester, one at 6–9 gestational weeks and the other at 11–14 gestational weeks. Gestational age was determined based on the date of the last menstrual period (LMP). If the difference was more than 5 days, gestational age was estimated based on ultrasound criterion at 6–9 gestational weeks provided that it was confirmed by ultrasound at 11–14 weeks. In our practice, at least two ultrasound examinations are routinely performed in the first trimester in all pregnant women, one at 6–9 gestational weeks and the other at 11–14 gestational weeks. All ultrasound examinations were performed by a perinatologist or radiologist experienced in obstetric sonography. Gestational age (GA) was determined by measuring of the fetal crown-rump-length (CRL) at 6–9 and 11–14 gestational weeks. The average CRL measurement in mm was derived from three satisfactory images. CRL was converted to the equivalent number of gestational days according to Hadlock *et al*. (1992). All pregnant women received their antenatal care at our hospital.

The collected data were maternal age, parity, neonatal weight, and neonatal complications. The following items were considered to evaluate the neonatal outcomes: transient tachypnea of the newborn (TTN) defined as the presence of tachypnea within hours after birth; respiratory distress syndrome (RDS) defined as the signs of respiratory distress (radiological features and oxygen therapy), sepsis, need for NICU hospitalization, and first minute and fifth minute Apgar scores.

### Statistical analysis and sample size

The sample size was calculated on the assumption that the incidence of TTN would be 3% in women with a delivery time between 38 and 39 gestational weeks and 1% in women with a delivery time ≥ 39 gestational weeks. The estimated sample size was 866 women in each group (α = 0.05; 1 - β = 0.80).

Categorical and continuous variables are expressed as number (percentage) and mean ± (standard deviation), respectively. Chi-square test was applied to compare categorical variables. Student’s t-test was used to compare parametric continuous variables. In order to control the potential confounders, a multiple logistic regression model was fitted for each dependent outcome variable (TTN, RDS, sepsis and need for NICU hospitalization). Explanatory variables were considered into the model for adjustment in the following order: maternal age, parity, neonatal weight, first minute Apgar, and fifth minute Apgar. The results are presented as odds ratios (OR) with 95% confidence intervals. Data analysis was undertaken using the Stata statistical software, released 13.0 (Stata Corp, College Station, Tex, USA).

## Results

Totally, 4892 CS were performed in our hospital during the study period, and based on the inclusion and exclusion criteria, 2086 patients were enrolled. In our study, 1002 (48%) women delivered between 38 and 39 gestational weeks and 1084 (52%) women delivered after 39 gestational weeks. Indications for elective caesarean delivery were prior caesarean section in 54.5% (1137 women), breech presentation in 7.2% (150 women), suspected cephalopelvic disproportion in 4.6% (96 women), maternal requested CS in 28.8% (601 women), and other causes such as retinopathy or myopathy and a history of infertility in 4.9% (102 women).

The mean ± (SD) age of the mothers who delivered between 38 to 39 gestational weeks was 27.77 ± (5.29) years, which was significantly higher than the mean (SD) age of mothers who delivered at > 39 gestational weeks (27.27 ± 5.74 years) (*P* = 0.041) (Table 1).

**Table 1:** Comparison of demographic characteristics of participants

Parameters	Elective caesarean delivery between 38 and 39 weeks gestation (*n* = 1002)	Elective caesarean delivery after 39 weeks gestation (*n* = 1084)	*P*-value
Maternal age (year)	27.77 ± (5.29)	27.27 ± (5.74)	0.041
Parity	1.89 ± (0.70)	1.90 ± (0.73)	0.675
Cause of cesarean			
Repeat cesarean	648(64.7)	489(45.1)	< 0.001
Breach and transverse presentation	42(4.2)	108(10)	
Cephalopelvic Disproportion (CPD)	38(3.8)	58(5.4)	
Maternal request	217(21.7)	384(35.4)	
Other reasons	57(5.7)	45(4.2)	
Birth weight of neonate	3190.77 ± (394.19)	3327.54 ± (377.70)	< 0.001
Height of neonate	49.57 ± (1.93)	50.17 ± (2.02)	< 0.001
Head circumference	34.47 ± (1.46)	34.59 ± (1.90)	0.113
Apgar min 1	8.97 ± (0.21)	8.99 ± (0.14)	0.033
Apgar min 5	9.99 ± (0.10)	10.00 ± (0.06)	0.529
NICU admission	22(2.2)	8(0.7)	0.004
Sepsis	6(0.6)	4(0.4)	0.329
Respiratory distress syndrome	7(0.7)	3(0.3)	0.141
TTN	15(1.5)	5(0.5)	0.013

Values given as mean ± SD(standard deviation), or number (percentage) unless otherwise indicated

As shown in Table 1, repeated caesarean was more frequent in mothers who delivered between 38 and 39 gestational weeks compared to mothers who delivered after 39 gestational weeks (64.7% vs. 45.1%, *P* < 0.001). Inversely, the frequency of breech presentation (10% vs. 4.2%), Cephalopelvic Disproportion (CPD) (5.4% vs. 3.8%), and maternal request (35.4% vs. 21.7%) were higher in mothers who delivered after 39 weeks’ gestation compared to mothers who delivered between 38 and 39 gestational weeks (P < 0.001).

The weight of neonates born after 39 gestational weeks was significantly higher than neonates born between 38 and 39 gestational weeks (mean difference: 136.76, 95% CI: 103.61 to 169.91, P < 0.001). Also, the one-minute Apgar score was significantly different between the two groups (mean difference: 0.02, 95% CI: 0.002 to 0.04, *P* = 0.029). There was no statistical significant difference in other characteristics between the two groups .

No significant differences were found in the incidence of neonatal sepsis between the two groups (0.6 and 0.4% in neonates born between 38 and 39 and after 39 gestational weeks respectively, adjusted OR: 1.60, 95% CI: 0.48 to 5.31, *P* = 0.440). The incidence of respiratory distress syndrome (RDS) was 0.7% (7 neonates) and 0.3% (3 neonates) in group 1 and 2, respectively. (Adjusted OR: 2.07, 95%CI: 0.56 to 7.56, *P* = 0.270).

The incidence of NICU admission was 2.2% (22 people) in neonates born between 38 and 39 gestational weeks and 0.7% (8 people) in those born after 39 gestational weeks. The difference was statistically significant (adjusted OR: 2.59, 95%CI: 1.16 to 5.79, *P* = 0.020).

The incidence of TTN was 1.5% (15 neonates) in neonates born between 38 and 39 gestational weeks (95% CI: 0.74 to 2.25) and 0.5% (5 people) in neonates born after 39 gestational weeks (95% CI: 0.05–0.08). The adjusted odds ratio of the association between TTN and time of delivery was 2.91 (95%CI: 1.09–7.76) (*P* = 0.032) using multiple logistic regression analysis, indicating that the minimal risk of TTN in neonates born between 38 and 39 gestational weeks was 9% higher compared to neonates born after 39 gestational weeks. Table 2 shows crude and adjusted odds ratio of the association between adverse outcomes and time of delivery.

**Table 2:** Crude and adjusted odds ratio (OR) for relationship between adverse outcomes and time of delivery

Variables	Crude OR	95% CI for Crude OR	Adjusted^a^ OR	95% CI for Adjusted OR
NICU admission	2.90	1.31–6.42	2.59	1.16–5.79
Respiratory distress syndrome	2.32	0.65–8.30	2.07	0.56–7.56
TTN	3.08	1.16–8.18	2.91	1.09–7.76
Sepsis	1.56	0.46–5.22	1.60	0.48–5.31

^a^ Adjusted for age of mothers, parity, weight of neonate, Apgar 1 min, Apgar 5 min

## Discussion

This study showed that elective CS delivery between 38 to 39 gestational weeks is associated with a higher rate of TTN and NICU admission in comparison with elective CS performed after 39 completed gestational weeks. Other major complications and more serious disorders such as respiratory distress syndrome (RDS) and sepsis were not significantly different between the two groups.

Matsuo et al. reported severe neonatal respiratory complications (RDS and TTN) that were similar in 415 Japanese pregnant women who underwent scheduled cesarean delivery in 38 and 39 gestational weeks [6]. In a study of 442,596 South Asian and Black women, Balchin et al. found that the rate of respiratory dysfunction was the lowest in white infants whose mothers underwent C-section after 39 gestational weeks and in South Asian infants whose mothers underwent C-section at 38 gestational weeks [7]. Moreover, Trata et al. examined 1951 cases of elective cesarean delivery in Japan and reported that elective cesarean should be performed at 38 weeks and two days or later in order to avoid respiratory complications [8].

A retrospective study of 1221 singleton pregnant women in Taiwan and Southeast Asia with scheduled cesarean delivery at 38 gestational weeks compared to 39 weeks revealed no statistically significant difference in severe neonatal complications including TTNB, RDS and NICU admission [1]. In contrast, the rate of NICU admission was higher in our study at 38 gestational weeksthan 39 gestational weeks. This difference may be attributed to differences in the hospital strategy to admit infants to NICU or high frequency of TTN.

Furthermore, most studies on Caucasian and mainly white women have emphasized the improved neonatal outcome in scheduled cesarean deliveries at 39 gestational weeks [9, 10]. It has been suggested that difference in the prevalence of respiratory dysfunction at different gestational ages in white and Asian infants can be due to genetic differences when the fetus matures in the uterine [7, 11]. Black and Asian infants have mostly shown meconium-stained amniotic fluid, indicating prematurity [11].

On the other hand, a multicentre clinical trial in Denmark showed that NICU admission was higher in scheduled cesarean delivery at 38 gestational weeks than those born at 39 gestational weeks,even though the difference was not significant [5]. It can be concluded that race alone cannot determine neonatal complications at different pregnancy ages. The difference of these studies conducted in the Caucasian and Asian communities in terms of the gestational age at the time of scheduled cesarean delivery can be due to differences in the sample size, failure to control confounding variables, or race.

In this study, the main reason for NICU admission was TTN, not other serious complications including RDS and sepsis. NICU admissions may lead to a large financial burden. Christopher J. Robinson et al. reported that waiting until 39 gestational weeks to perform an elective CS is cost effective [12]. However, the likelihood of emergency cesarean delivery and its maternal complications should also be taken into account. As suggested by some studies, the mean gestational age in Asian and black populations is less than the whites, which can be due to fetal prematurity [11]. Emergency cesarean can be followed by complications for mothers and infants [13]. Thus, in our study, we cannot draw a definite conclusion that delivery should be performed after 39 gestational weeks. Further studies are required to compare the rate and complications of emergency and scheduled cesarean delivery in Iranian population at 38 and 39 gestational weeks.

One of the concerns that causes scheduled cesarean delivery at 38 gestational weeks is prevention of unexpected fetal death. In this study, we did not have any cases of stillbirth. The risk of unexplained stillbirth at 38 gestational weeks was reported to be about 0.05 per 1000 births among women with the history of cesarean delivery in a Scottish and a Canadian cohort study [14, 15]. To measure the prevalence of 0.01–1% with sufficient accuracy and confidence, as many as 20,000 cases are needed [16]. Therefore, a small size of the sample may be the reason.

In this study, the most common causes of cesarean delivery in the two groups were a previous history of CS and maternal request. However, the frequency of repeated cesarean delivery was higher at 38–39 gestational weeks than its frequency after 39 gestational weeks. Two-thirds of births were done at 38–39 gestational weeks for this reason. Moreover, 56% of all repeated cesarean deliveries were performed before 39 gestational weeks. A multicentric cohort in the United States showed that one-third of cesarean deliveries are performed before 39 gestational weeks. The rate is between 50 and 80% in some European countries. The reason for this concern is maternal complications in pregnancy associated with previous CS [17]. In this study, other reasons, including maternal request, breech presentation, and CPD, were more prevalent at 39 gestational weeks.

In our study, almost half of elective caesarean sections were performed before 39 gestational weeks. Wilmink et al. reported that more than 50% of the elective cesarean sections were done before 39 gestational weeksin the Netherlands (8.3% at 37 gestational weeks and 48.3% at 38 gestational weeks [10]. Zanardo V. et al. reported that about 60% of elective cesarean sections in their hospital were performed before 38 weeks and 6 days [18] It seems that physicians, regardless of the existing documents, perform a large percentage of CS deliveries before 39 gestational weeks. It may be due to the physician’s opinion or practice patterns, maternal request for early CS, birth certificate data, or any other reason.

The strong points of this study were excluding the cases of emergency cesarean delivery and a large sample size. The limitations of this study were evaluating neonatal complications only up to 28 days after birth not beyond 28 days.

## Conclusion

According to our study findings, scheduled CS delivery between 38 and 39 gestational weeks is associated with a higher rate of NICU admission in comparison with scheduled CS performed after 39 gestational weeks. The main reason for NICU admission was TTN, not other serious complications. Although no cases of intrauterine fetal death occurred in women who underwent CS delivery after 39 gestational weeks, we did not have any information on the incidence and complications of emergency CS in those who delivered after 39 weeks’ gestation.Therefore, based on the results of this study, it cannot be concluded that scheduled caesarean section should be performed after 39 gestational weeks. We suggest that the above issue be examined in the future studies to determine the time of scheduled CS more accurately.

## Abbreviations

CPD: Cephalopelvic Disproportion
CRL: Fetal crown-rump-length
CS: Cesarean sections
GA: Gestational age
LMP: The last menstrual period
OR: Odds ratios
RDS: Respiratory distress syndrome
TTN: Transient tachypnea of the newborn
